# Comparative impact of streptozotocin on altering normal glucose homeostasis in diabetic rats compared to normoglycemic rats

**DOI:** 10.1038/s41598-023-29445-8

**Published:** 2023-05-16

**Authors:** Habib ur Rehman, Kaleem Ullah, Aamir Rasool, Robina Manzoor, Yu Yuan, Abdul Malik Tareen, Imdad Kaleem, Naveeda Riaz, Sahir Hameed, Shahid Bashir

**Affiliations:** 1 Department of Microbiology, University of Balochistan, Quetta, 87300 Pakistan; 2 Institute of Biochemistry, University of Balochistan, Quetta, 87300 Pakistan; 3 Faculty of Marine Sciences, Water and Marine Sciences, Lasbella University of Agriculture, Uthal, 90150 Pakistan; 4 College of Life Sciences, North China University of Science and Technology, 21 Bo Hai Road, Tangshan, 063210 People’s Republic of China; 5 Department of Biosciences, COMSATS University Islamabad, Islamabad, Pakistan; 6 Department of Biological Sciences, International Islamic University, Islamabad, Pakistan; 7 National Institute of Genomics and Advanced Biotechnology (NIGAB) National Agriculture Research Council (NARC), Islamabad, Pakistan; 8 Neurosciences Center, King Fahad Specialist Hospital Dammam, P.O. Box 15215, Dammam, Saudi Arabia

**Keywords:** Biotechnology, Cell biology, Endocrinology

## Abstract

Diabetes mellitus is a syndrome and an endocrine disorder, primarily considered as a loss of glucose homeostasis because of the insulin action and/or secretion or both. Currently there are more than 150 million people in the world affected by diabetes mellitus with a higher share of Asian and European countries. The current study aimed to investigate the comparative altering properties of streptozotocin (STZ), based on up-turn and down-turn configuration of biochemical, toxicological and hematological parameters in comparison with normoglycemic male albino rats. This comparative study was conducted among normoglycemic and STZ based induced-type 2 diabetic male albino rats groups. The male albino rats were intra-peritoneally injected with STZ with the dose rate of 65 mg/kg body weight for one time to developed type 2 diabetic model. Biochemical (blood glucose, uric acid, urea and creatinine), toxicological (AST, ALT and ALP) and hematological parameters (red and white blood cells) and their functional indices were evaluated in type 2 diabetic induced group along with normoglycemic rats. The STZ based induced- type 2 diabetic rats showed statistically significance (p < 0.001) higher level in the blood glucose, alongwith the change in the levels of biochemical parameters including urea, uric acid, and creatinine. Toxicological parameters comprising AST, ALT and ALP were also shown significance (p < 0.001) as sufficient after experimental evaluation of biologically important parameter in STZ based induced-type 2 diabetic rats. Likewise, the red blood cells, white blood cells and their efficient components were exposed significantly insufficient after the injecting of STZ to induce the rats as type 2 diabetic. The results of the current study indicates the comparatively higher levels of variation among biochemical, toxicological and hematological parameters in STZ based Induced-type 2 diabetic model as compared to normoglycemic group.

## Introduction

Diabetes mellitus/ hyperglycemia is a metabolic disease seriously effective from defects in insulin secretion and/or insulin action on receptor locus and is mostly considered by raised blood glucose levels. The hereditary and physical circumstances both are linked in the pathological process of diabetes mellitus. It is identified as the 3rd highest serious syndrome globally^1^. Diabetes mellitus is primarily divided into two types, such as type 1 (insulin dependent) and type 2 (non-insulin dependent). Body is unable to synthesize sufficient insulin in case of type 1 diabetes mellitus while type 2 diabetes mellitus has a characteristics of development of resistance for insulin hitting and this insulin dependency aspect is temporary. The epidemiological data has declared that Currently there are 246 million people are infected with diabetes mellitus globally and this amount is assumed to be raised up to 300 million by the year 2030^2^. Several different pathogenic aspects are assumed to be frequently associated with the development of severe diabetes mellitus. These factors include age, obesity, heredity, ethnicity, reduced physical mobility, immune disorders, defects in pancreatic beta cells and insulin resistance^3^. Mainly causes associated with Type 1 diabetes include genetic disorders and autoimmune destruction of pancreatic beta cells^4^. Type 2 diabetes is primarily due to heightened glucose production and glucagon secretion along with peripheral insulin resistance in the body^5^. Long-term consequences of poorly controlled diabetes include chronic kidney disease, retinopathy and diseases of the heart and arterial circulation^6,7^.

Several validated scientific and effective research has been conducted for searching and testing the new antidiuretic drugs with standardization by developing several models of animal for examining diabetes mellitus. The several animals models has been developed via surgical operations (pancreatectomy), fed a diet or nutrients-based diabetes induction in animals, and through genetic-based operations (spontaneous and transgenic/knockout diabetic animals) in different animal species to persuade both the types of diabetes. However, the best and greater communal technique for developing diabetes in research-based animals is the practicing of diabetogenic preparations containing the streptozotocin (STZ) with or without nicotinamide, alloxan monohydrate, dithizone, ferric nitrilotriacetate and several additional compounds^8^.

On behalf of the different studies, STZ is reflected as the maximum utilised diabetogenic chemical for persuading diabetes in research animals. STZ exists as *N*-nitrosoamide derivatives extracted from *Streptomyces acromogenes*. STZ induces irreversible necrosis of cells randomly and rapidly through pancreatic-selective-cell toxigenic effect. STZ has selective toxic effects on β-cells of pancreatic islets because of high affinity for β-cell membrane, low capacity of β-cells to scavenge free radicals, and low NAD + /DNA ratio in islets. Due to accumulation of STZ in pancreatic β-cells by Glut2, other organs like kidney, liver, and intestine are also damaged by STZ^9^. Moreover, the STZ capability to develop insulin-dependent diabetes mellitus (type 1) by thorough elimination or devastation of pancreatic cells, the STZ is moreover having the capability of producing peripheral insulin resistance (type 2 diabetes), impairing the glucose oxidation or impairing insulin secretion, decreasing the insulin synthesis and secretion from β-cells. STZ also disrupts the glucose transport and glucokinase activity. Such effects are mainly and sufficiently lead to develop noninsulin-dependent diabetes mellitus (type 2) in animals^10^.

Various results specified that creating mild to severe pattern of diabetes is depend upon the vary and selected dose of STZ, strain, animals age, dietetic status, and administration route, accompanied by further factors^11^. Therefore, scholars are appreciated to organize their particular procedures of developing type 2 diabetes through STZ in accordance with specific research necessities.

The phase or status of inducing the type 2 diabetes in animals is particularly arbitrated by attaining a condition of hyperglycemia that defines an upsurge in the measured glucose standard point after STZ injection. Usually, rats possessing primary glucose range above 200 mg/dL is reflected hyperglycemic and is incorporated in studies. The effect of difference in blood glucose levels resulting type 2 diabetes developments in rats may possibly show the existence of various stratum of glucose homeostasis interference, which may perhaps influence the outcomes of checking antidiabetic reagents^12^.

## Materials and methods

### Chemical

Diabetogenic drug Streptozotocin (STZ) of Sigma Chemicals, Germany was purchased and used in the current study.

### Animals

Albino male rats, of 10 to 12 weeks age, weighing 150 to 200 g each, were obtained from DUHS Karachi for the present study. The rats were housed and maintained in animal house of University of Balochistan Quetta under controlled room temperature and humidity with required light–dark cycles for the purpose of controlled supervision on Rats. All experiments were approved and performed in accordance with relevant institutional licensing committee guidelines and regulations (Ethics committee of Animal and Human of University of Balochistan Quetta). The study was carried out in compliance with the ARRIVE guidelines. The rats were admitted free approach to standard diet and water ad libitum. All the standard protocol in-accordance with GLP (Good Laboratory Practice) Regulations of WHO was followed (Elina et al. 2015). Fasting blood glucose (FBG) level was measured after fasting overnight using Glucometer (AccuChek Active Performa, Roche, Germany) via tail vein to confirm the normal fasting blood glucose level. The day of confirmation of normal FBG level was considered as day 4 of experiment.

### Experimental design and induction of type 2 diabetes

The male albino rats were distributed into two (02) groups randomly. Group (C1) was kept as control. Type 2 Diabetes was induced in group (D1) by injecting the STZ^®^ (Sigma Chemicals, St. Louis, MO) @ 65 mg/kg Body weight (single dose) intraperitoneally, prepared in citrate buffer of 4.5 pH after overnight fasting state. Fasting blood glucose (FBG) level was measured using Glucometer (AccuChek Active Performa, Roche, Germany) 3 days (72 h) after the administration of STZ^®^ to confirm induction of diabetes. Rats having blood glucose level 220–250 mg/dl were considered as diabetic. The day of confirmation of induction of diabetes was considered as day 4 (passing 72 h after) of experiment.

### Experimental procedure

The regular analysis of fasting blood glucose (FBG) was measured on (0, 4, 7) days and (7, 9) weeks respectively using Glucometer (AccuChek Active Performa, Roche, Germany) responsible for blood glucose determining after developing of diabetes, while all the rats were kept fasted for overnight. After passing over 8 weeks of the trial, the rats were humanely euthanized, the blood was taken directly from the heart of albino male rats and preserved in anticoagulant coated vacutainer (10% of ethylene diamine tetracetic acid -EDTA) for determination of hematology including white blood cell count (WBC), red blood cell count (RBC), hemoglobin concentration (Hb), hematocrit (Hct), mean corpuscular volume (MCV), mean corpuscular hemoglobin (MCH), mean corpuscular hemoglobin concentration (MCHC), platelet (Plt) and differential white blood cell count. Vacutainer lacking anticoagulant were exposed to centrifuge at 1500 rpm for 15 min for isolation of serum. The serum was preserved at − 20 °C for more investigation.

### Serum analysis

Liver function markers (AST, ALT, ALP, TOTAL BILIRUBIN, GGT) and Kidney function markers (Creatinine, Urea, Total Protein, A.G Ratio) were evaluated through advanced commercially accessible diagnostic kits.

### Statistical analysis

The data was analyzed as mean ± standard error of mean (SEM), statistically the groups considered as independent and parameters as dependent variables. Analysis of variance (Oneway-ANOVA), unpaired *t* test were used while using SPSS software. The P-value < 0.001(***) was considered statistically extremely significant, P-Value < 0.01 (**) was measured statistically very significant and NS = Not significant. Statistical significance was set as p < 0.05.

## Results

### Streptozotocin (STZ) activity and Blood glucose level

Statistical analysis revealed that streptozotocin caused extremely significant (p < 0.001) induced type 2 diabetes in experimental albino rat models by specific cytotoxicity action on pancreatic β-cells, led to affect the endogenous insulin release/action or both, resulted an increased in the fasting blood glucose (mg/dl) and glycosylated hemoglobin (HbA1c) levels measured on (0, 4, 7) days and (7, 9) weeks respectively using Glucometer (AccuChek Active Performa, Roche, Germany) in STZ treated/type 2 diabetic group (D1) as contrasted to untreated/control group (C1) as presented in Table 1.

**Table 1:** Mean ± SEM values of fasting blood glucose level (mg/dl) and glycosylated hemoglobin (HbA1c) (%) in Control (C1) and Diabetic (D1) rat groups induced with STZ @ 65 mg/kg b.w once.

Fasting blood glucose (FBG) (mg/dL)						
Group	Group abbre	Day 0	Day 4	Day 7	Week 07	Week 09
Control	C1	92 ± 0.84	89 ± 0.51	94 ± 0.77	87 ± 0.58	91 ± 0.85
Diabetic	D1	96 ± 0.49	341 ± 1.67	440 ± 1.99	524 ± 2.05	579 ± 3.26
P value		***	***	***	***	***
Glycocylated Hb (%)		5.68	11.96	12.89	13.47	14.49

***p < 0.001 (extremely statistically significant).

### Hematological parameters

Statistical analysis revealed that chronic diabetes caused extremely significant (p < 0.001) reduction in hematological parameters (WBCs, MCV, MCH and MCHC) along with Neutrophils and Eosinophil and very significant reduction pattern (p < 0.01) in Packed cell volume. The Red blood cell (RBC) and Hemoglobin (Hb) were noticed as not statistically significant (NS) b/w the two groups. The elevated pattern of Platelet count with (p < 0.001) along with up-turn in percentage of Lymphocyte and Monocytes was significantly measured in treated/type 2 diabetic group (D1) as distinguished to untreated/control group (C1) as presented in Table 2.

**Table 2:** Mean ± SEM values of comparative Hematological parameters in Control (C1) and Diabetic (D1) rat groups.

Hematological values			P value
Parameters	Control (C1)	Diabetic (D1)	
RBC (× 10^6^ cells/mm^3^)	9.32 ± 0.51	8.12 ± 0.29	NS
PCV (%)	55.12 ± 0.68	53.21 ± 0.25	**
Hb (g/dl)	16.32 ± 0.22	16.1 ± 0.08	NS
WBCs (× 10^3^/mm^3^)	6.46 ± 0.13	5.19 ± 0.05	***
MCV ( μm3)	56.54 ± 0.15	54.5 ± 0.08	***
MCH (Pg)	19.12 ± 0.04	18.19 ± 0.10	***
MCHC (g/dL)	34.5 ± 0.02	33.08 ± 0.12	***
Platlets (× 10^5^ cells/mm^3^)	612 ± 22.05	696 ± 7.78	***

Differential white blood cells (%)			
Parameters	Control (C1)	Diabetic (D1)	
Lymphocytes	71.09	82.61	
Monocytes	1.19	2.03	
Neutrophils	27.54	15.56	
Eosinophil	1.34	1.03	

***p < 0.001 (extremely statistically significant).

**p < 0.01 (very statistically significant).

*NS* not statistically significant.

### Liver and kidney profile measurement

Mean ± SEM values of comparative altered serological parameters of Liver and Kidney profiles in control and type 2 diabetic groups at different stages like (stage 0 (< 200) = day 0 (control), stage 1(220–490) = day 7(diabetic), stage 2(491–600) = week 9(diabetic) respectively. In comparison of control group vs. Type 2 diabetic group (Day 07) and control group vs. Type 2 diabetic group (week 09), a statistically extremely significant (p < 0.001) elevated pattern were noticed in the concern parameter of AST, ALT, ALP, GGT, Creatinine, Blood urea, Uric acid, Globulin, Fibrinogen and Total Bilirubin (Direct Bilirubin and Indirect Bilirubin), and extremely significantly (p < 0.001) declined level of Albumin and A.G Ratio in both type 2 diabetic stages e.g., stage 1(220–490) = day 7(diabetic) and stage 2(491- 600) = week 9(diabetic) as compared to normal “stage 0 (< 200) = day 0 (control)” group as shown in Table 3. Statistically not significant (p < 0.2) measurements was recorded in the “Total protein” obtained from both type 2 diabetic stages (stage 1and stage 2) as compared to normal (control) stage as revealed in Table 3.

**Table 3:** Mean ± SEM values of comparative altered serological parameters in different stages (stage 0 (< 200) = day 0 (control), stage 1 (220–490) = day 7 (diabetic), stage 2 (491–600) = week 9 (diabetic).

Fasting Blood Glucose (mg/dL) →	Stage 0 (< 200)	Stage 1 (220–490)	Stage 2 (491–600)	P value	
	Day 0	Day 07	Week 09		
Parameters	Control	Diabetic	Diabetic	Control vs. diabetic day 07	Control vs. diabetic week 09
Albumin (g/dL)	4.6 ± 0.08	4.1 ± 0.10	3.8 ± 0.06	**	***
Globulin (g/dL)	2.1 ± 0.11	3.1 ± 0.06	4 ± 0.02	***	***
A.G ratio	2.2 ± 0.03	1.32 ± 0.07	0.95 ± 0.13	***	***
Urea (mg/dL)	41.1 ± 0.9	72.8 ± 3.16	146.7 ± 82.93	***	***
Creatinine (mg/dL)	0.6 ± 0.004	1.3 ± 0.01	1.7 ± 0.01	***	***
Uric acid (mg/dl)	2.64 ± 0.02	5.27 ± 0.06	7.92 ± 0.14	***	***
Total protein	6.7 ± 0.41	7.2 ± 0.06	7.8 ± 0.62	NS	NS
ALT (IU/L) SGPT	38.4 ± 0.16	89.5 ± 5.9	81.3 ± 2.6	***	***
AST (IU/L) SGOT	44.1 ± 1.47	133.3 ± 7.60	153.4 ± 7.36	***	***
ALP (IU/L)	91.7 ± 10.37	249 ± 11.86	319.5 ± 7.96	***	***
GGT (IU/L)	2.1 ± 0.20	3.7 ± 0.30	9.1 ± 1.23	**	***
Fibrinogen (mg/ml)	3.34 ± 0.18	4.98 ± 0.10	5.49 ± 0.11	***	***
Total bilirubin	0.58 ± 0.00	1.42 ± 0.01	1.79 ± 0.00	***	***
Direct bilirubin	0.26 ± 0.00	0.66 ± 0.00	0.88 ± 0.00	***	***
Indirect bilirubin	0.32 ± 0.00	0.76 ± 0.01	0.91 ± 0.00	***	***

***p < 0.001 (Extremely statistically significant).

**p < 0.01 (Very statistically significant).

*NS* not statistically significant.

## Discussion

Diabetes mellitus (DM), impaired glucose homeostasis, is the frequently arising endocrine disorder that leads to the dysfunctional characteristics of multisystem in the body. Systemic and metabolic disturbances due to diabetes ultimately results in hyperglycemia. The biomolecules are effected in the diabetes which leads to produce free radicals, impairing the antioxidant patterns in the different system of the body^13^. Streptozotocin is well-known as diabetogenic chemical agent to induce the both types of diabetes mellitus in experimental animal models by specific cytotoxicity action on pancreatic β-cells leads to affect endogenous insulin discharge/action or both resulting an increase in the fasting blood glucose level^14^. STZ extremely significantly (p ≤ 0.001) had increased effect on the serum glucose and glycosylated hemoglobin measurements in diabetic group. This research was planned to investigate the progressive hematological findings of male albino rats induced to type 2 diabetic by Streptozotocin. In addition, the effect of diabetes was also analyzed on liver and kidneys.

In this experimental study, STZ-induced type 2 diabetic rats were categorized by a significant upsurge in their blood glucose level along with their constituents measurement in contrast with the control rats as mentioned in several prior research experiments^15^. The induced levels of blood glucose and glycosylated hemoglobin were keenly statistically observed at extremely significantly higher level in the present STZ-induced type 2 diabetic study which is consistent with the previous reports^16^.

Hematological outcomes had led us to the not significant (NS) pattern of RBCs and Hb values. Anemic condition in type 2 diabetes might be associated with increased non-enzymatic glycosylation of RBCs by reason of lipid peroxidation. These lasting diabetogenic alterations were in same pattern to the Erukainure et al.^17^ and Usman et al.^18^. There is statistically significant downfall in the concentration of hematocrit, MCV, MCH and MCHC in the current experiment which is analogous to the results of previous study^19^. There is a great role of Platelets or thrombocytes in the clotting of blood by senthesizing a fibrin fiber meshwork at the place of vascular opening and prevent the blood loss^20^. The content of platelets was significantly elevated with an increased significant measured of the percentage of Lymphocyte and Monocytes in the type 2 diabetic rats when correlated to control/normal rats with Bedoya et al.^21^. Raised platelet count in diabetic patients may perhaps autonomous presumption of insulin resistance. Rising of platelet count leads to raise the insulin resistance which further leads to undesired effect against cardiovascular function^22^.

Body immunity leads to suppress by administration of STZ follow the establishing of type 2 diabetes. This significant decrease in WBCs count may be associated to the vulnerable leukocytosis from the bone marrow which may give explanation for deprived self-protective processes in contrast to infection^20^. Neutrophils and Eosinophil had been observed to be significantly lower the normal values during experiment in type 2 diabetic group as compared to control one, while there was significantly increased tendency of Lymphocytes and Monocytes in experimental rats as compared to normal, which is in accordance with Akomas et al.^23^.

As the diabetes is a metabolic disorder, leads to cause harm to hepatocytes. Due to hepatic cells injury, directly linked with the discharge of intracellular elements into the systemic circulation. Hepatic cells damage can be scientifically diagnosed or a valuable clue may be easily provided by the measurement of serum concentrations of hepatic enzymes. Induction of type 2 diabetes mellitus with STZ directed statistically a significant (p < 0.001) increase in the Liver and Kidney profiles activity of serum aspartate aminotransferase (AST), alanine aminotransferase (ALT), alkaline phosphatase (ALP), Creatinine, GGT, Blood urea, Uric acid, Globulin and Fibrinogen in both type 2 diabetic stages e.g., stage 1(220–490) = day 7(diabetic) and stage 2(491–600) = week 9(diabetic) in the type 2 diabetic rats in comparison to normal ”stage 0 (< 200) = day 0 (control)” group as said the normal/control group. A significantly (p < 0.001) raised pattern in the level of serum bilirubin in the current research also showed the experimental induction of type 2 diabetes. The elevated pattern of serum bilirubin level mainly due to the reduction of liver uptake, the more formation of bilirubin or conjugation. A significant (p < 0.001) reducing was noticed in the Albumin and A.G Ratio parameters obtained from both type 2 diabetic stages (stage 1and stage 2) as compared to normal (control) stage. The increased levels of hepatic enzymes may be due to discharge of enzymes from the hepatic tissue in the plasma in STZ-induced type 2 diabetes. The results of current study are according to the earlier research analysis^16,24^. Insulin deficiency causes an increase in catabolism of skeletal muscle which causes an increase breakdown of protein that leads to an increase of total protein in the blood^25^. In our results, the total proteins were found not significantly (p < 0.2) increased in both type 2 diabetic stages (stage 1and stage 2) as compared to normal (control) stage as same as revealed in Nazki et al.^26^.

## Conclusion

In conclusion, the current research work has potentially revealed the diabetogenic effectiveness of STZ to induce the hyperglycemia by enhancing the beta cells apoptosis and to improve the results consequences in the proper scientific control of complications linked with type 2 diabetes in the course of further research. The current diabetogenic hematological parameters investigation also supports the improvement of various hepatological and nephrological constituents in the agreement of high level research studies.

## Data Availability

The datasets used and/or analysed during the current study available from the corresponding author on reasonable request.
